# Overcoming resistance against managed care – insights from a bargaining model

**DOI:** 10.1186/s13561-017-0156-4

**Published:** 2017-05-22

**Authors:** Andree Ehlert, Thomas Wein, Peter Zweifel

**Affiliations:** Leuphana University of Lueneburg, Scharnhorststr. 1, Lüneburg, 21335 Germany

**Keywords:** Managed care, Game theory, Multilateral Nash bargaining, Health insurance, Consumer choice, Healthcare reform, Germany, The Netherlands, Switzerland, I13, I11, D02, C72

## Abstract

Recent healthcare reforms have sought to increase efficiency by introducing managed care (MC) while respecting consumer preferences by admitting choice between MC and conventional care. This article proposes an institutional change designed to let German consumers choose between the two settings through directing payments from the Federal Health Fund to social health insurers (SHIs) or to specialized MC organizations (MCOs). To gauge the chance of success of this reform, a game involving a SHI, a MCO, and a representative insured (RI) is analyzed. In a “three-player/three-cake” game the coalitions {SHI, MCO}, {MCO, RI}, and {SHI, RI} can form. Players’ possibility to switch between coalitions creates new outside options, causing the conventional bilateral Nash bargaining solution to be replaced by the so-called von Neumann-Morgenstern triple. These triples are compared to the status quo (where the RI has no threat potential) and related to institutional conditions characterizing Germany, the Netherlands, and Switzerland.

## Background

In several countries reforms have been undertaken during the past years to increase efficiency, and in particular, to contain healthcare expenditure. Examples are Denmark, Germany, Norway, Spain, and the United States (see e.g. [8, 9, 16, 26]). Attempts were also made to improve the matching of healthcare supply with consumer preferences (consumer-driven health care, see [6]).

However, as soon as consumers are given a choice of insurer (or provider) it is not sufficient to model healthcare reform as the outcome of a process involving the government (a social health insurer, SHI, respectively) and an organization representing healthcare providers. Unless the reform proposals are in accordance with the preferences of a third player, namely the insured, taxpayers, and patients, they are bound to fail. Typical examples are the failed attempt to impose cost sharing on the Dutch (likely because they are exposed to substantial financial risk through reduced short-term disability payments, see [15]), to get the Germans to sign up for managed care (MC, known as “Integrated Care", see [10]), and to get the Swiss to accept MC rather than fee-for-service combined with free physician choice as the default option [28]. Since most of current reforms involve MC, consumer choice essentially is between conventional care (CC, often fee-for-service) and MC, which is often outsourced to specialized managed care organizations (MCOs).

This paper contains a proposal designed to strengthen the influence of individual citizens in the provision of health care. Taking the case of Germany, it suggests that citizens rather than their health insurer obtain the right of deciding whether conventional providers or MCOs receive payment from the so-called Federal Health Fund (FHF). For simplicity, it is assumed that both the SHI and the MCO have the resources to “buy off” the medical association and the hospital association, who therefore do not figure as separate players. Our main assumptions are that the reform proposal permits both the SHI and the MCO to set up MC plans in independent bilateral agreements with the insured (or to form a coalition against the insured) and that MC plans are financially viable. Thus, in game-theoretic terms, each pair of players bargains over one of three cakes (in general of different size) representing efficiency gains through MC. Note, however, that MC will not be the dominant outcome due to SHI’s intrinsic preference for CC.

To gauge the chances of success for this proposal, the Nash bargaining solution [18] is adapted to the “three-player/three-cake" situation [4]. In contrast to bilateral bargaining, this solution is primarily determined by outside options, i.e. breaks of existing coalitions and renegotiations of either party with the third player. To our knowledge this is the first application of multilateral Nash bargaining in the context of health economics (for applications of bivariate Nash bargaining to health economics, see e.g. [5, 11, 21]). While multilateral Nash bargaining theory has been applied to more general economic issues (see e.g. [1, 25]), nontrivial objective functions characterizing the players have rarely been considered for the three-player/three-cake problem.

The remainder of this text is structured as follows. The “Institutional background” section contains a description of the institutions governing the provision of health care in three insurance-based countries, Germany, the Netherlands, and Switzerland. These three countries allow a degree of consumer choice in health care, which however is hampered by cartelization of health insurers and service providers. In the “Methods” section a bargaining model is developed that first involves the SHI association and a MCO in a bivariate setting, representing the status quo. The reform proposal then introduces a representative insured (RI) as a third player. In the “Results” section several solutions of the game based on specific parameter constellations are studied. A generalization of the results to the Netherlands and Switzerland is provided in the “Discussion” section. The “Conclusion” section summarizes the paper. A list of main symbols (in order of appearance) is provided in Appendix A. Technical details are discussed in Appendices B to D.

## Institutional background

Invariably, healthcare reform involves at least two players. One is an organization representing consumers and taxpayers. In insurance-based systems, this is a social health insurer (or an association of regulated health insurers where choice is permitted). In National Health Service-based systems, this is the government. The other player is an organization representing healthcare providers. The most powerful usually is the national medical association. However, in a country that is strongly hospital-oriented, this could also be the association of (public) hospitals. Modelling these two players is sufficient in countries where citizens are tied to a social health insurer through their professional status (as e.g. in Austria) or to a regional healthcare system (as e.g. in Sweden or Norway).

By way of contrast, there are countries where consumers can choose between competing health insurers. In Europe, the Netherlands and Switzerland can be seen as blueprints for this type of system [3, 20, 22]. In Switzerland, policyholders have free choice between health insurers who charge lower premiums for higher deductibles and MC policies [13]. There is no involvement of employers, making health insurance a true consumer choice. Depending on the canton of residence, a subsidy kicks in at a premium amounting to eight to ten percent of taxable income, while public welfare pays the premium for the very poor. All variants of MC, ranging from “soft” second-opinion programs to “harsh” Health Maintenance Organizations (HMOs), are offered by at least some (competing) SHIs. In a popular referendum held in 2012, Swiss voters rejected a bill that would have made MC rather than conventional fee-for-service the standard of health care [28]. Nevertheless, the market share of MC (mainly the “soft” variants) had attained almost 50% by 2010.

The 2005/6 reform in the Netherlands made citizens sign a contract with a health insurer (just continuing with the current insurer was disallowed), either non-profit or for-profit. The core universal insurance package is financed by a payroll tax paid by the employer (50%), a premium which must be independent of age or status of health (45%), and general taxation (5%). Higher deductibles than the minimum of EUR 150 per year can be selected in exchange for a premium reduction. MC alternatives (Preferred Provider Organizations and HMOs) are available, but are rarely chosen [24]. Summing up, the Dutch and Swiss healthcare systems have several features of managed competition: insurer-specific, non-discriminating premiums, risk adjustment schemes to prevent cream skimming, and premium reductions for (higher) deductibles and MC alternatives.

**Fig. 1:**
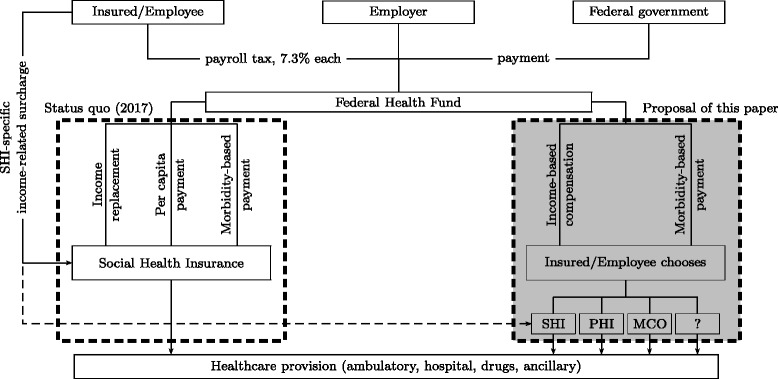
Financial structure of German social health insurance as of 2017 (*left-hand side*) and reform proposal (*right-hand side*)

In Germany, competition was injected into the healthcare system by the Health Care Structure Reform Act of 1996. Earners of incomes below EUR 37,000 (as of 1996) obtained the right to sign up with a SHI of their choice (those with incomes above this limit always had this right, in addition to opting for private health insurance, PHI). In spite of risk adjustment based on age, gender, average labor income, and rate of unemployment, contribution rates drifted apart. Since politicians were not willing to attribute this development to differences in efficiency, they pushed for an increased amount of redistribution between SHIs. In 2009, this resulted in the creation of the FHF. Effective 2015, insured and employers pay an equal payroll tax (7.3%) into the FHF, which also receives a contribution from the federal government (see Fig. 1). However, the SHIs obtained the right to impose firm-specific surcharges on the payroll tax in order to avoid a deficit. At the same time, these surcharges serve as major elements of competition between SHIs in addition to minor differences in service. Variations in contributions due to income differences between insured populations are compensated by the FHF.

With the potential exception of a surcharge, SHIs do not receive contributions from their members anymore. Rather, they obtain a per-capita payment from the FHF which varies slightly as a function of age, sex, and labor income. This payment is designed to finance one-half of insurers’ healthcare expenditure (HCE). The other half is governed by morbidity-based risk adjustment, which distinguishes 80 clusters of high-cost diseases. Patients are assigned to these clusters by the attending physician, who takes also into account HCE caused by long-term drug use.

Since patients sometimes renege on the MC obligation to obtain treatment from within the network, payment for services provided out-of-network needs to be arranged. A law promulgated in 2011 rules that the insurer receives avoided cost from the FHF, meaning that it is reimbursed the HCE which would have been incurred inside the MC network. Since the value of this HCE is not known when contribution rates are set, the individual’s previous HCE is used as a proxi, which however may be too low in the case of deteriorating health. Two solutions to this problem have been discussed. One is to econometrically predict HCE for the 80 clusters of diseases. The other is to allow the SHI to negotiate the avoided cost payment directly with physicians. According to [7], this is preferable.

An issue peculiar to Germany is that earners of high incomes and independent workers can opt out of SHI in favor of PHI. In the present context, this raises two points. For one, many policyholders cannot afford to switch between private insurers after a few years because the new insurer would charge a much higher premium. Second, high-income individuals can avoid the redistribution inherent in SHI which is seen as social injustice by many. Hence, [12] propose that everyone, independent of insurance status, pay the payroll tax. In return, private insurers would receive reimbursement from the FHF as well. This proposal comes close to creating an individual right to decide who receives one-half of one’s contribution to health insurance.

The institutional innovation proposed and analyzed in this paper is to give individuals the right to assign the full FHF payment not only to the SHI or the PHI of their choice but also to a MCO – as well as any other recipient capable of organizing healthcare services (cf. the question mark in Fig. 1).^1^ The only requirement is that they must be able to provide the full range of services, viz. ambulatory, hospital, drugs, and ancillary. Otherwise, one would run into the unsolvable problem of splitting HCE in advance. Conversely, new entrants into the market must have non-discriminatory access to physicians, hospitals, drugs, and ancillary services. As in deregulated access to electricity and gas grids as well as in international trade, rules of nondiscrimination need to be enforced if hitherto closed, cartelized markets, are to be opened.

## Methods

This section is devoted to a game-theoretic analysis designed to find out whether the reform proposal advanced at the end of the preceding section has a chance to succeed. It starts out with the situation prevailing before 1996 (see the “State no. 1: no consumer choice” section). Next, the status quo where the SHI decides whether it wants to outsource MC service provision to a MCO is modeled in two steps (see the “State no. 2: the status quo” and “State no. 3: flexible prices” sections). Finally, the game is complemented by a representative consumer as a third player (see the “State no. 4: introducing consumer choice” section). As stated in the “Institutional background” section neither the (regional) medical association nor the (national) hospital association appear in the game, although both are very powerful in Germany. This simplification can be justified by the fact that both the SHI and the MCO dispose of the resources for “buying off” healthcare providers. As to the SHIs, fees negotiated with the service providers have always satisfied their participation constraints: On the one hand, German medical faculties continue to face excess demand, causing them to impose a numerus clausus; on the other hand, exits of hospitals are an extremely rare event. As to the MCOs, they stand to achieve cost savings (see the “State no. 2: the status quo” section) which they can use to overcome the status quo preference of physicians (which is substantial judging from Swiss experience [27]). Therefore, provider interests are taken care of by either player.

### State no. 1: no consumer choice

Consider first a SHI scheme à la Bismarck with all residents having compulsory insurance of the same type. There is no choice of insurer or plan as was the case in Germany prior to the Reform Act of 1996. Let there be a homogeneous population of $\bar {N}$ insured. Insurers are non-profit public bodies aiming at reputation and/or market share, i.e.

1 $$ u_{SHI} = u_{SHI}(\underset{(+)}{\mathstrut N_{CC}},\underset{(+)}{\mathstrut B}).  $$

Here, *u*_*SHI*_ formalizes SHI’s objective function, where $N_{CC}\le \bar N$ denotes the number of insured under CC, and *B* represents a budget available for public relations e.g. in order to enhance the SHI’s profile. One may limit the analysis to one insurer as Germany is characterized by regional SHI monopolies. The following assumptions are made.

#### **Assumption 1**

- There is no consumer sovereignty concerning choice of plan. The insurer may arbitrarily allocate insured to either MC or CC, for whom MC and CC offer equal quality of care.
- The insurer receives a payment *π* per representative insured from the FHF that exactly covers its costs for patient treatment and administration. There are no payments from outside the system. Consequently, the positive impact of *N*_*CC*_ on the insurer’s utility is entirely based on reputation.
- The only way for the insurer to generate a financial surplus is by shifting a number of $N_{MC} = \bar N - N_{CC}$ insured to an insurer-owned MC plan. The rent per insured is *μ*_*SHI*_≥0 which reflects average savings e.g. through innovative modes of treatment.

To see that under Assumption 1 the SHI’s optimal level of MC may be low, consider a simple parametric example of (1) where *N*_*CC*_ and *N*_*MC*_ are substitutes, i.e.

2 $$ u_{SHI,1}= N_{CC}^{\alpha} (\mu_{SHI}N_{MC})^{\beta},\quad \alpha,\beta>0.  $$

Here, *μ*_*SHI*_ *N*_*MC*_=*B* represents insurer surplus. The optimal level of MC is attained at

3 $$ N_{MC}^{*} = \frac{\beta}{\alpha+\beta} \bar N  $$

which may be a low number for large values of the parameter *α* relative to *β*, i.e. for an insurer mainly interested in its head count.

### State no. 2: the status quo

In order to reflect policy efforts designed to encourage MC in several European healthcare systems beginning in the 1990s, state no. 2 is characterized by professional MCOs that specialize in designing MC plans. They achieve higher cost savings and hence higher surplus per insured than SHI, i.e. *μ*_*MCO*_≥*μ*_*SHI*_.

Assumption 1 continues to hold since there is no consumer sovereignty regarding the choice between CC and MC. As quality of care is assumed to be the same, the insured are indifferent between plans as long as contributions are identical.^2^ In contrast, the “State no. 4: introducing consumer choice” section contains a model with the insured as an active player who exercises his or her right to choose between CC and MC.

The SHI acts as a gatekeeper with respect to MC for two reasons. First, all insured are initially enrolled in CC, which is typical of early stages of MC development. Second, by Assumption 1a, the insurer holds the exclusive right to payments from the FHF. Consequently, the MCO must negotiate with SHI over prices and quantities for a transfer of insured. The market thus becomes a bilateral regional monopoly with one “seller” of insured (SHI), and one “buyer” (MCO).

Three games are considered. First, let the MCO and SHI play a non-cooperative game involving the number of insured that SHI supplies and MCO demands at a predetermined price *μ*_*SHI*_≤*p*≤*μ*_*MCO*_.^3^ This situation reflects early-stage MC markets where prices are rigid or even regulated and where there is little trust between players. The second game allows for cooperation while prices are still fixed. Third, an efficiency-enhancing negotiation over both prices and quantities is considered in the “State no. 3: flexible prices” section.

To formally describe the first game, some additional notation is required. Let the strategy of MCO consist of *N*_*MC*,*D*_, i.e. the number of insured that MCO plans to contract with, and let SHI choose both *N*_*MC*,*S*_ (the number of insured it is prepared to transfer to MCO at a given price *p*) and *N*_*SHI*,*MC*_ (the number of insured in SHI’s own MC plan). Note that two MC plans (one for MCO and one for SHI) may exist sidy-by-side even though *μ*_*SHI*_≤*μ*_*MCO*_.

For a given “demand” *N*_*MC*,*D*_, SHI’s objective function (2) needs to be modified to read, with subscript *nc* for “non-cooperative”,

4 $$ \begin{aligned} &u_{SHI,nc}(N_{SHI,MC}, N_{MC,S})= \\ &\qquad (\bar N-N_{SHI,MC} -N_{MC,S})^{\alpha} (N_{SHI,MC}\mu_{SHI}\\ &\qquad + p\min\{ N_{MC,D}, N_{MC,S}\})^{\beta}. \end{aligned}  $$

Here, $\bar N - N_{SHI,MC} - N_{MC,S} = N_{CC}$ reflects the residual number of insured to be served in the CC setting. Further, for a given “supply” *N*_*MC*,*S*_, the MCO as a profit maximizer is characterized by

5 $$ \begin{aligned} & u_{MCO,nc}(N_{MC,D})= \\ &\qquad (\mu_{MCO} - p) \min\{N_{MC,D}, N_{MC,S}\}. \end{aligned}  $$

Note that the minimum operators in (4) and (5) reflect the fact that equality of supply and demand may not be guaranteed in a non-cooperative setting. In particular, the SHI bears the risk that *N*_*MC*,*D*_<*N*_*MC*,*S*_ in which case its reputation is damaged (first term in (4)), while only effective demand *N*_*MC*,*D*_ valuated at *p* is compensated (second term in (4)). For this reason the strategy $N_{MC,S}(N_{MC,D}) = N_{MC}^{*}$ and *N*_*SHI*,*MC*_=0 does not constitute an equilibrium (in light of the threat *N*_*MC*,*D*_=0 or small). From (4) and (5) one can derive the following reaction functions (for details, see Appendix B),

6 $$  N_{SHI,MC}(N_{MC,D}) = N_{MC}^{*} - N_{MC,D} \frac{\alpha p + \beta\mu_{SHI}}{(\alpha+\beta)\mu_{SHI}}  $$

for $0 < N_{MC,D} \le \frac {\bar N\beta \mu _{SHI}}{\alpha p + \beta \mu _{SHI}}$, and

$$N_{SHI,MC}(N_{MC,D}) = 0, \text{otherwise}. $$

Therefore, the SHI may decide not to offer its own MC plan at all (*N*_*SHI*,*MC*_(*N*_*MC*,*D*_)=0) if the MCO plans to insure people beyond a certain limit. This limit is the lower, (1) the smaller the total population ($\bar N$), (2) the less surplus matters to the SHI (*β*), (3) the more the number of conventionally insured matters to the SHI (*α*), and (4) the higher the price charged by the MCO for taking over an insured (*p*).

7 $$\begin{array}{*{20}l} N_{MC,S}(N_{MC,D}) &= \left\{\begin{array}{ll} N_{MC,D} & \quad\text{for~} 0 \le N_{MC,D} \\ &\quad \le N_{MC}^{*}, \\ N_{MC}^{*} & \quad\text{for~} N_{MC,D} > N_{MC}^{*}; \end{array}\right. \end{array} $$

8 $$\begin{array}{*{20}l} N_{MC,D}(N_{MC,S}) &= \;\;\, N_{MC,S} \quad\text{for~} N_{MC,S} \ge 0  \end{array} $$

where $N_{MC}^{*}$ is determined by (3).^4^ These two reaction functions together determine whether the supply of and the demand for MC-insured are in equilibrium. Supply increases in response to demand *N*_*MC*,*D*_ as long as it does not exceed an optimum $N_{MC}^{*}$ (from the point of view of the SHI). This optimum is the higher, (1) the larger the total population ($\bar N$), (2) the more surplus matters to the SHI (*β*), and (3) the less the number of conventionally insured matters to the SHI (*α*). When it is reached, supply does not respond to demand anymore. Conversely, demand for MC-insured by the MCO increases in step with the supply offered by the SHI.

**Fig. 2:**
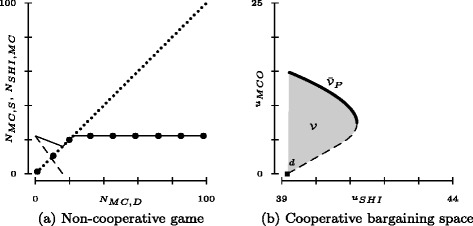
Panel (**a**) shows a non-cooperative game over MC-insured. *Big dots* represent the reaction function *N*_*MC*,*S*_(*N*_*MC*,*D*_), the *dashed line*, *N*_*SHI*,*MC*_(*N*_*MC*,*D*_), and *small dots*, *N*_*MC*,*D*_(*N*_*MC*,*S*_). The *solid line* represents the overall number of MC-insured given by *N*_*MC*,*S*_+*N*_*SHI*,*MC*_. Panel (**b**) shows the cooperative bargaining space $\mathcal {V}$ (*shaded area*) with its Pareto frontier $\bar {\mathcal {V}}_{P}$ (*solid*). The threat point *d* is marked with a *solid square*

Illustrative reaction functions are depicted in Fig. 2 a, which exhibits a continuum of Nash equilibria, i.e. *N*_*MC*,*S*_=*N*_*MC*,*D*_ for $0\le N_{MC,D} \le N_{MC}^{*}$. The following parameter values are used, which are retained throughout unless otherwise specified ($\kappa, \bar {c}$ and *ρ* will be explained below):

9 $$ \begin{aligned} \mu_{SHI} &= 1, \alpha = 0.7, \kappa = 1, \bar c = 5, \\ \mu_{MCO} &= 2, \beta = 0.2, \rho=2, \bar N = 100,\\ p &=(\mu_{SHI}+\mu_{MCO})/2 = 1.5 \end{aligned}  $$

Note that the relatively small value of *β* compared to *α* represents the SHI’s assumed focus on head count instead of its financial status.

In Fig. 2 b, instead, SHI and MCO cooperatively determine *N*_*MC*_ (where the indices *S* or *D* are dropped in view of their cooperation). The objective functions read, with subscript *c* denoting “cooperative”,

10 $$\begin{array}{*{20}l} u_{SHI,c} &= (\bar N - N_{SHI,MC} - N_{MC})^{\alpha}\\ &\qquad (pN_{MC} +\mu_{SHI}N_{SHI,MC}- \bar c)^{\beta}  \end{array} $$

11 $$\begin{array}{*{20}l} u_{MCO,c} &= (\mu_{MCO} - p) N_{MC}- \kappa \bar c  \end{array} $$

where bargaining is assumed to involve additional costs $\bar c$ for both SHI and MCO. To take into account the possibility of higher initial bargaining costs for MCOs (due to their lack of market experience) a scaling factor *κ*≥1 appears in (11).

Associated with each admissible bargain $0\le N_{MC}\le \bar N$ is a pair (*u*_*SHI*,*c*_(*N*_*MC*_, *N*_*SHI*,*MC*_), *u*_*MCO*,*c*_(*N*_*MC*_)) of payoffs, with $N_{SHI,MC}\le \bar N - N_{MC}$ chosen independently by SHI. The set of these values lie to the northeast of $d= (d_{SHI}, d_{MCO}) = (u_{SHI,1}(N_{MC}^{*}),0)$, spanning the bargaining space $\mathcal {V}$ (see Fig. 2 b).^5^ The threat point *d* is attained in case of disagreement (with no bargaining cost charged by assumption). It reflects the fact that MCO is unable to secure positive utility without the consent of SHI whereas the latter can independently attain at least $u_{SHI,1}(N^{*}_{MC}) >0$ by choosing $N_{SHI,MC} = N^{*}_{MC}$. For later reference, define $g_{i}: \mathbb R_{+} \to \mathbb R_{+}$ as player *i*’s maximum payoff in $\mathcal V$ for a fixed payoff *t* of player *j* (and *g*_*j*_ accordingly). More precisely, let

12 $$ g_{i}(\mathcal V, t) = \left\{\begin{array}{lll} \max\{x:(x,t)\in \mathcal V \} &\quad \text{if~} (0,t)\in\mathcal V,\\ 0 &\quad \text{otherwise}. \end{array}\right.  $$

The intersection of the graphs of *g*_*i*_ and *g*_*j*_ yields $\bar {\mathcal V}_{P}$, the Pareto-efficient boundary of $\mathcal V$. For example, using (10) and (11) the Pareto frontier $\bar {\mathcal V}_{P}$ is attained for

13 $$ \begin{aligned} &N^{*}_{MC} + \frac{\alpha \bar c }{p(\alpha+\beta)} \le N_{MC} \le \bar N \quad \text{and}\\ &N_{SHI,MC}=0. \end{aligned}  $$

Note that comparing (13) to (7) and (8) one finds that all non-cooperative Nash equilibria are inefficient. In particular, SHI cannot obtain $N^{*}_{MC}$ in this case (except for the limiting case $N^{*}_{MC}$ with *α*=0 or $\bar c=0$). Economically, the shift from a non-cooperative to a cooperative solution amounts to a shift away from market share and towards financial status as the main determinant of SHI utility.

In a von Neumann-Morgenstern sense the whole set $\bar {\mathcal V}_{P}$ represents the cooperative solution of the game. In this article, the Nash bargaining solution [18] is used to single out a particular point on $\bar {\mathcal V}_{P}$. This solution is based on a set of axioms that have become widely accepted, portraying a notion of fairness (cf. [19]). Formally, the (unique) point $v = v(\mathcal V,d)$ that maximizes (*x*_1_−*d*_1_)(*x*_2_−*d*_2_) subject to $x \in \mathcal V$ is determined. Evidently, $v\in \bar {\mathcal V}_{P}$. Let $\tilde {N}_{MC}$ denote the corresponding value of the bargain *N*_*MC*_. Note that no closed form expression exists for $\tilde N_{MC}$ for objective functions such as (10) and (11). Using (9), one obtains the numerical solution $\tilde N_{MC} = 29.4$ and *v*=(41.0,9.7).

In the remainder of this section, this model is applied in an attempt to explain why recent political efforts to increase the level of *N*_*MC*_ in Germany and Switzerland (at least as far as the “harsh” variant is concerned) have largely failed while in the Netherlands, gatekeeping by primary care physicians has become the standard. First, returning to the non-cooperative game, note that MCO’s increasing demand *N*_*MC*,*D*_ in early stages of MC markets comes along with a temporary decline in the total number of insured in MC (see the solid line in Fig. 2 a for small values of *N*_*MC*,*D*_). The reason is that SHI’s low productivity in MC provision causes the number *N*_*SHI*,*MC*_ of insured in SHI’s own MC plan to be disproportionately reduced as soon as a lucrative opportunity (*p*>*μ*_*SHI*_) to outsource MC production to MCO emerges (cf. (4) and (5)). More formally, differentiating (6) yields

$$\frac{\partial N_{SHI,MC}}{\partial N_{MC,D}} = -\frac{\alpha p + \beta \mu_{SHI}}{(\alpha+\beta)\mu_{SHI}}\le -1 $$

for $0\le N_{MC,D}\le \frac {\bar N\beta \mu _{SHI}}{\alpha p + \beta \mu _{SHI}}$. Note, however, that this argument does not apply to the Dutch variant of MC. There, prices for healthcare services are still largely regulated by law, preventing insurers from outsourcing their MC activities. In addition, there has been no clear separation between CC and MC (at least in terms of contractual arrangements between insurers and service providers) in the Netherlands even before 2006, resulting in the absence of a distinct MC start-up phase (see [20]).

At a time when MC in the guise of gatekeeping was firmly established (around 2000, say), it was still in early development in Switzerland and especially in Germany. In both countries, MCOs were in fact specialized divisions of SHIs or joint outsourced operations that were managed without much cooperation with traditional CC divisions. With policy makers unable to change either the SHI’s or the MCO’s objective function, lowering transaction cost is their only instrument involving e.g. start-up funding, reducing legal uncertainty about contract design, and supporting advertising of MC.

Turning to the cooperative game, one would expect a reduction of transaction cost $\bar c$ to also make a difference. However, evaluation of $\frac {\partial \tilde N_{MC}}{\partial \bar c}$ is complicated by the lack of a closed-form expression for $\tilde N_{MC}$. Yet it is possible to determine the sign of this derivative using the concept of equivalent threat points (see e.g. [19]). More precisely, there exists an alternative threat point *d*_*a*_=(0,*x*) such that $ v(\mathcal V, d)= v(\mathcal V, d_{a}) $ where *x* is unknown (but given (10) and (11), *x*≥0). Under the new threat point *d*_*a*_, maximization of the Nash product^6^

$$\big((\bar N - N_{MC})^{\alpha} (p N_{MC} - \bar c)^{\beta}\big) \big((\mu_{MCO} - p)N_{MC} - \kappa \bar c - x \big) $$

yields a standard quadratic equation that is solved for

14 $$ \tilde N_{MC} = \sqrt{\eta^{2}-\theta} - \eta  $$

with

$$\begin{array}{*{20}l} \eta &= \frac{p\bar N (1+\beta) + \bar c(1+\alpha)}{2p(1+\beta+\alpha)} \\ \theta& = \frac{c\bar N}{p(1+\alpha + \beta)} + \frac{(\kappa \bar c + x)(\bar N \beta p + c\alpha)}{p(\mu_{MCO}-p)(1+\alpha+\beta)}. \end{array} $$

Now, from (14) it follows that $\frac {\partial \tilde N_{MC}}{\partial \bar c} > 0$ for values of *x*≥0 and *κ*≥1. This result is intuitive. The higher fixed transaction cost, the larger the break-even number of insured that need to be transferred to MC for negotiation to be successful. It is interesting to see that the splitting of the cost between the two players is not relevant.

### State no. 3: flexible prices

In this section bargaining occurs over both *N*_*MC*_ and *p*, with *μ*_*SHI*_≤*p*≤*μ*_*MCO*_, while SHI continues to choose *N*_*SHI*,*MC*_ independently. To describe $\bar {\mathcal V}_{P}$ for flexible prices it is necessary to consider two cases. In the first case, $\bar {\mathcal V}_{P}$ is given by (10) with

15 $$\begin{array}{*{20}l} N_{MC} &= \frac{\alpha (u_{MCO} + \bar c (1+\kappa)) + \beta \bar N \mu_{MCO}}{(\alpha + \beta)\mu_{MCO}}  \\ p &= N_{MC} - \frac{u_{MCO} + \kappa \bar c}{N_{MC}} \\ N_{SHI,MC} &= 0, \end{array} $$

for 0≤*u*_*MCO*_≤*u* ^∗^. Here,

$$\begin{array}{*{20}l} u^{*}&= \left(\frac{(\mu_{MCO} - \mu_{SHI}) [\alpha \bar c (\kappa + 1) + \beta \bar N\mu_{MCO}]}{(\alpha + \beta)\mu_{MCO}} - \kappa \bar c\right)  \\ &\qquad \left(1 - \frac{(\mu_{MCO}-\mu_{SHI})\alpha}{(\alpha + \beta)\mu_{MCO}}\right)^{-1}. \end{array} $$

In the second case, one has $u^{*} < u_{MCO}\le (\mu _{MCO}-\mu _{SHI}) N -\kappa \bar c$, *N*_*SHI*,*MC*_=0, and

$$\begin{array}{*{20}l} N_{MC} &= \frac{u_{MCO} + \kappa \bar c}{\mu_{MCO} - \mu_{SHI}}  \\ p &= \mu_{SHI}.  \end{array} $$

For details, see Appendix C.

**Fig. 3:**
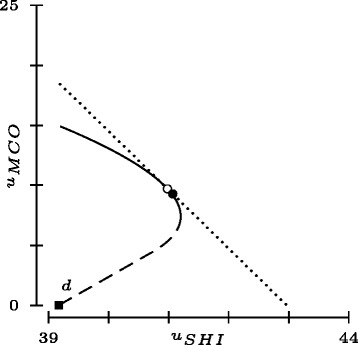
The *dotted frontier* reflects bargaining over both *N*_*MC*_ and *p* with solution (41.1, 9.3) indicated by the *solid circle*. The *solid square* represents the threat point where, as before, $d_{SHI}=u_{SHI,1}(N_{MC}^{*})=39.2$ and *d*_*MCO*_=0. For comparison, the *solid/dashed frontier* corresponds to bargaining over *N*_*MC*_ with *p* fixed (as in Fig. 2 b), with solution (41.0, 9.7) indicated by the *open circle*

Figure 3 shows that freely negotiable prices are efficiency-enhancing, i.e. the new bargaining set (dotted boundary) contains the former bargaining set for state no. 2 (boundary with solid and dashed segments). In fact, efficient trades with low values of *u*_*MCO*_ are characterized by relatively high prices (cf. (15)). Incidentally, Fig. 3 provides an example for a well-known criticism of the Nash solution. It is not monotonic in the sense that $v(\mathcal V', d) \ge v(\mathcal V,d)$ for $\mathcal V\subseteq \mathcal V'$ (cf. [17]). In this example, the MCO loses out when the bargaining set is expanded. This risk may explain why Swiss SHI who had MCO divisions or had jointly created a MCO did not oppose an ordinance limiting the premium reductions for MC plans to 20% of the CC alternative although achievable cost savings were higher. Evidently, another explanation is that a constraint of this type serves as a coordinating device in an oligopoly facilitating joint profit miximization.

### State no. 4: introducing consumer choice

Up to this point, it was sufficient to consider two players, SHI and MCO. However, this paper proposes to give consumers rather than the SHI the right to choose between CC or MC, cf. the “Institutional background” section. Due to the assumed equality of CC and MC in terms of quality, player RI is indifferent between the two. Therefore, money *σ* is the only argument in the utility function of RI,

16 $$ u_{RI}= u_{RI}(\sigma) = \sigma^{1/\rho},\qquad\rho\ge 1,  $$

with 1−1/*ρ*≥0 denoting RI’s coefficient of relative risk aversion. For consumers to be able to choose between CC and MC, they must have the right to channel their contribution to either SHI or MCO (in the case of Germany, they would designate the FHF payment accordingly). Therefore, the assumptions of the “State no. 1: no consumer choice” section need to be changed as follows.

#### **Assumption 2**

- The consumer RI has full sovereignty concerning choice between SHI and MCO (where there is an obligation to choose). As before, both plans offer equal quality of care.
- SHI (MCO, respectively) receives payment *π* per insured (in the case of Germany, from FHF) that exactly covers its costs for patient treatment and administration.
- As before.

A fundamental difference between this setup and the game in the “State no. 2: the status quo” section is the ability of any two of the three players to form a coalition and to implement a MC plan independently of the third player’s action. There is no cake for an individual player {RI}, {SHI}, {MCO} or for the grand coalition {SHI,MCO,RI}; in the latter case, bargaining costs are assumed to be prohibitive.

In contrast to the “State no. 2: the status quo” and “State no. 3: flexible prices” sections, the SHI loses its unconditional right to obtain a contribution (the refund from the FHF in the case of Germany) and hence its gatekeeping position by Assumption 2a.^7^ Still, coalition {SHI,MCO} might form, which renders RI’s right to choose either SHI or MCO worthless in view of compulsory insurance, thus undermining the whole idea of RI sovereignty.

The notion of a market for MC-insured where supply (SHI) and demand (MCO) are negotiated in exchange for some payment *p* is no longer appropriate. Instead, players bargain over the number of insured they control (supplemented by side payments, if applicable). Formally, the partition $\bar N = \bar N_{SHI} + \bar N_{MCO} + \bar N_{RI}$ splits the number of insured under the respective player’s control.^8^ As the game provides no cake for single players, one has $\bar {N}_{k}=0$ (player *k*) in case of *i* and *j* forming coalition {*i* *j*}. The objective functions for this bargaining situation are given by (16) and

17 $$\begin{array}{*{20}l} u_{SHI,4} &= (\bar N_{SHI} - N_{SHI,MC})^{\alpha} \\ &\qquad (\mu_{SHI} N_{SHI,MC} - \bar c - \sigma + \omega)^{\beta}  \end{array} $$

18 $$\begin{array}{*{20}l} u_{MCO,4} &= \mu_{MCO} \bar N_{MCO} - \kappa \bar c - \sigma -\omega  \end{array} $$

where *σ*≥0 denotes money to compensate RI for his or her cooperation in a coalition {SHI, RI} or {MCO, RI}. The amount *ω*>0 represents a side payment within coalition {SHI, MCO} raising the possibility to trade money in addition to the number of insured. Here, SHI faces the sub-partition $\bar N_{SHI}= N_{SHI,MC} + N_{SHI,CC}$, where *N*_*SHI*,*CC*_ represents the number of insured in CC, and *N*_*SHI*,*MC*_ the number of insured in SHI’s MC plan. Note that each player in a bilateral coalition has the outside option to bargain alternatively with the remaining third player. The solution to this “three-player/three-cake” bargaining [4] is given by the unique outcome vector with the property that no player can improve upon its outcome by switching coalitions.

To formally state this solution, define first a constrained bilateral Nash bargaining game that takes the existence of an outside option into account. In a second step, the outside options are determined endogenously.

The solution to the constrained Nash bargaining game (denoted $\tilde v$ to distinguish it from the unconstrained solution *v*) for two players *i* and *j* with predetermined outside option $m\in \mathbb R^{2}_{+}$ is given by $\tilde v(\mathcal V_{\{ij\}},d,m) = \arg \max _{x} (x_{i}-d_{i})(x_{j}-d_{j})$ for *x*≥*m* and $x\in \mathcal V_{\{ij\}}$, and *m* otherwise (see [2]). Intuitively, outside options give rise to lower bounds for the player’s payoff.

For the global game $(\mathcal V_{\{ij\}}, \mathcal V_{\{ik\}}, \mathcal V_{\{jk\}}, d)$ where *d*=(*d*_*i*_,*d*_*j*_,*d*_*k*_), the multivariate Nash bargaining solution is defined by the set $x = (x_{\{ij\}}, x_{\{jk\}}, x_{\{ik\}})\in \mathbb R^{2\times 3}$ where $x_{\{ij\}} = \tilde v(\mathcal V_{\{ij\}},d, m_{\{ij\}}(x))$. Here, $m_{\{ij\}}\in \mathbb R^{2}$, with $m_{\{ij\},i}(x) = g_{i}(\mathcal V_{\{ik\}},x_{\{ki\},k})$ and $m_{\{ij\},j}(x) = g_{j}(\mathcal V_{\{jk\}},x_{\{kj\},k})$. The notation {*i* *j*},*i* points to player *i* within coalition {*i* *j*}, and analogously for the sets {*j* *k*} and {*i* *k*}. The outcome *x* represents a (unique) set of reciprocal conjectures about bargains in all coalitions (for technical details, see [2, 4]). Note that this outcome comprises some rather degenerate cases where e.g. due to a dominant coalition the multivariate solution boils down to the (constrained) bivariate Nash solution (see [2] for a detailed characterization in terms of the “strength” of coalitions). The primary solution, however, arises when the game is relatively symmetric in the sense that the core is empty.^9^ In this case the outcome *x* corresponds to a von Neumann-Morgenstern (VNM) triple.

#### **Definition 1**

*([* 4 *])* The set *x*=(*x*_{*ij*}_) for all coalitions {*i* *j*} is a VNM triple if each *x*_{*ij*}_ belongs to $\bar {\mathcal V}_{P,\{ij\}}$, and *x*_{*ij*},*i*_=*x*_{*ik*},*i*_ for all *i*.

It is shown in [4] that a multivariate Nash bargaining solution always exists for the three-player/three-cake problem and that it is unique. This does not imply, however, that the solution corresponds to a certain bilateral coalition.

The solution answers two types of questions: What coalitions might form? If a certain coalition forms, what will be the proposed outcome? When the core is empty, these answers may appear unsatisfactory because the VNM triple constitutes an infeasible vector of outcomes comprising all bilateral coalitions. This apparent weakness is in the very nature of cooperative solutions. However, the VNM triple has the advantage of being independent of starting conditions and the order of players (see [4] for a discussion). When a “strong” coalition exists, however, the multivariate bargaining outcome may be a definite number pertaining to this coalition (cf. [2] for details). Two such examples are analyzed in the “Results” section below.

## Results

Since Nash bargaining does not in general admit of closed solutions, the results of numerical simulations are exhibited in Table 1, with parameter values as in (9). The market share of MC (column *N*_*MC*_) is shown to increase slightly from state no. 1 (before reforms) to state no. 2 (SHI acting as gatekeeper, prices fixed), and on to no. 3 (flexible prices). The utility values for both players (*u*_*SHI*_,*u*_*MCO*_) do not change much either (where here and in the following we use, somewhat incorrectly, the term utility also for SHI’s and MCO’s objective functions).

**Table 1:** Utility values and MC market shares for bivariate Nash bargaining (no. 2 and 3) and multivariate Nash bargaining (no. 4)

	Bargaining state	Parameters	*u*_*SHI*_	*u*_*MCO*_	*u*_*RI*_	*N*_*MC*_
1.	no. 1		39.2	–	–	22.2
2.	no. 2		41.0	9.7	–	29.5
3.	no. 3		41.1	9.3	–	29.7
4.	no. 4: {SHI, MCO}	*ω*=0	12.6	128.3	–	78.0
5.	no. 4: {MCO, RI}	*ω*=0	–	128.3	8.2	100.0
6.	no. 4: {SHI, RI}	*ω*=0	12.6	–	8.2	78.0
7.	no. 4: {SHI, MCO}	*ω*>0	14.5	133.2	–	77.9
8.	no. 4: {MCO, RI}	*ω*>0	–	133.2	7.9	100.0
9.	no. 4: {SHI, RI}	*ω*>0	14.5	–	7.9	74.2
10.	no. 4: {SHI, MCO}	*μ*_*MCO*_=0.8; *ω*=0	21.2	30.6	–	60.7
11.	no. 4: {MCO, RI}	*μ*_*MCO*_=0.8; *ω*=0	–	30.6	6.7	100.0
12.	no. 4: {SHI, RI}	*μ*_*MCO*_=0.8; *ω*=0	21.2	–	6.7	60.7
13.	no. 4: {MCO, RI}	$\bar c=55$; *ω*=0	–	96.7	6.9	100.0
14.	no. 4: {SHI, RI}	*κ*=36; *ω*=0	25.1	–	5.8	52.5
15.	no. 4: {SHI, RI}	*μ*_*MCO*_=0.2; *ω*=0	25.1	–	5.8	52.5

Parameters are set according to (9) unless otherwise stated in the “Parameters” column

**Fig. 4:**
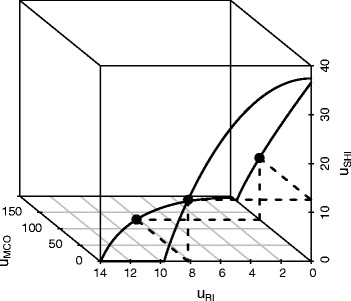
VNM triple (*solid dots* joined by *dashed line*) with bilateral Pareto frontiers $\bar {\mathcal {V}}_{P}$ (*solid*) for the “three-player/three-cake” game defined by (16) to (18) with *ω*=0

Rows 4 to 12 of Table 1 refer to the three-player/three-cake game in state no. 4 where using (16) to (18) a VNM triple turns out to exist. As stated in Definition 1 the triple specifies a unique fixed point, i.e. the sole outcome vector such that for each player bargaining in either of two possible coalitions is equivalent, cf. Fig. 4. The VNM triple may be obtained numerically by solving the system $g_{i}(\mathcal V_{\{ij\}}, x_{\{ij\},j}) = x_{\{ij\},i}$, $g_{i}(\mathcal V_{\{ik\}}, x_{\{ik\},k}) = x_{\{ik\},i}$, and $g_{k}(\mathcal V_{\{jk\}}), x_{\{jk\},j}) = x_{\{jk\},k}.$

The sets $\mathcal V_{\{ij\}}$ (or, equivalently, $\bar {\mathcal V}_{P,\{ij\}}$ by (12)) with *d*=(0,0,0) for the three coalitions {SHI, MCO}, {SHI, RI} and {MCO, RI} are derived in Appendix D.

Evidently, the SHI loses out, being unable to maintain its former utility values. In rows 4 to 6 where side payments between SHI and MCO are disallowed (*ω*=0) the market share of MC increases substantially, no matter which bivariate coalition becomes effective. The parties who stand to benefit are the MCO and (somewhat) the RI. From RI’s point of view it is interesting to note that unlike observed in Table 1 the increase in his or her payoff in state no. 4 is not necessarily linked to a higher value of *N*_*MC*_.^10^

If side payments are allowed (as in rows 7 to 9 where *ω*>0), the overall picture does not change much. It is interesting to note, however, that *N*_*MC*_ tends to decrease slightly. To see why recall that side payments between SHI and MCO are used to compensate the inefficiency inherent to SHI’s MC production (*μ*_*SHI*_≤*μ*_*MCO*_). Consequently, SHI chooses *N*_*SHI*,*MC*_=0 (see Appendix D). Now, MCO’s higher productivity creates leeway for SHI’s actually preferred option, namely to increase enrolment in CC (at least as long as *α*>*β*).

Rows 10 to 12 underline the fact that three player bargaining in state no. 4 assigns positive utilities even to inefficient MCOs with *μ*_*MCO*_<*μ*_*SHI*_. Of course, such a MCO loses out when compared to rows 4 to 6, with *N*_*MC*_ substantially lower (except for {MCO, RI}). The most striking finding, however, is that inefficient MCOs in state no. 4 may outperform efficient MCOs in states no. 2 and 3 (where inefficient MCOs would not even be able to enter the market). The reason is that by Assumption 2 player RI does not take efficiency of MC production into account in its choice.

In view of these findings, the SHI as the loser because of RI’s increased sovereignty might be tempted to establish a strong coalition, as in rows 13 to 15 of Table 1 e.g. by artificially increasing total per-person transaction cost $\bar c$ or MCO’s cost factor *κ* (note however that this would run against the intentions of policy makers, who seek to reduce transaction costs burdening MCOs). Row no. 13 exhibits the consequences of the first alternative. It actually backfires on SHI because the indeterminacy of the multilateral Nash solution is resolved in this case. The predicted coalition is {*M* *C* *O*,*R* *I*}, causing the payoff to SHI to drop to zero. However, row no. 14 shows that keeping $\bar c$ constant as in (9) while burdening the MCO with a higher share of it (*κ*=36 rather than *κ*=1) establishes the coalition {*S* *H* *I*,*R* *I*}.

Finally, the sensitivity of the values of the VNM vector (as in rows no. 4 to 6) with respect to marginal parameter changes is discussed. As before, algebraic expressions for the respective derivatives cannot be obtained.

**Fig. 5:**
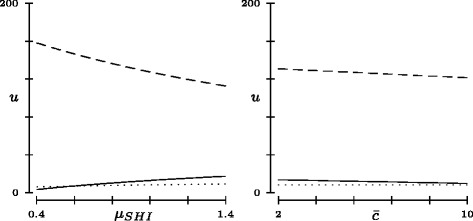
Sensitivity analysis. Utility values according to the multilateral Nash bargaining solution (state no. 4; *ω*=0) for players SHI (*solid line*), MCO (*dashed*) and RI (*dotted*) in relation to marginal changes in *μ*_*SHI*_ (*left panel*) and $\bar c$ (*right panel*). The remaining parameters are set to (9)

Figure 5 shows simulation results for the objective functions in state no. 4 (with *ω*=0). Here, the values of *μ*_*SHI*_ and $\bar c$ range within the indicated intervals, while the remaining parameters are set to (9). Results for SHI and MCO are as expected; yet note that increasing *μ*_*SHI*_ (SHI’s efficiency of MC provision) is also in the interest of RI (cf. the slight increase in the dotted line of the left panel). Further, a cost increase affects both SHI and MCO, but cannot be passed on to RI (horizontal dotted line of the right panel).

## Discussion

In this section, an attempt is made to link the bargaining models to Dutch and Swiss experience and to assess the likely outcome if the reform proposal for Germany of this paper were adopted. Recall that the Netherlands and Switzerland introduced RI as a player in 2005/6 and 1996, respectively. With the Dutch reform of 2005/6, consumers had to explicitly choose an insurer (the differentiation between SHIs and private health insurers had been lifted), with the option of selecting HMO as the “harsh” variant. So far, few have exercised it; experimental evidence [15] even suggests that the Dutch had positive willingness to pay for a return to free physician choice. Apparently, they do not judge CC and MC as equivalent in terms of quality, contrary to the basic assumption adopted in this paper. One way to relate this to the parameters of the model would be to let the efficiency advantage of MCO become very small or even negative once the cost of effort necessary to persuade consumers to accept HMO is taken into account (cf. row no. 15 in Table 1). Another way is to let the factor *κ* of (high) transaction cost falling on the MCO become big, as in row no. 14 of Table 1. Either way induces the coalition {SHI, RI}, resulting in exclusion of MCO.

In the case of Switzerland, a choice experiment revealed that the insured need to be compensated for attributes characterizing MC [29]. For instance, accepting a physician list based on cost criteria was found to require a reduction of CHF 103 in the monthly contribution to social health insurance, amounting to 38% of the country’s average premium. However, this estimate has a standard error (s.e.) of 13, indicating that a sizable share of the Swiss population may give up free physician choice for less compensation. In addition, the amount drops to CHF 42 (s.e. 7.8) if the list is based not only on cost but also quality criteria, as is the case with less harsh variants of MC such as physician networks. Indeed, by 2012 more than one-half of the insured had opted for MC, albeit mainly for physician networks rather than HMOs. Yet when parliament passed a bill that would have made MC rather than CC the default option in Swiss social health insurance, a popular referendum was called. A two-third majority of voters rejected the bill. In terms of the simulations, row no. 6 of Table 1 comes close to depicting developments since the 1996 reform. Because SHIs created MCOs themselves, consumers did not have an outside option that would have benefitted an individual MCO. Therefore, {SHI, RI} is the only possible coalition with a relatively high share “transferred” to an integrated MCO and SHI reaping the available surplus which it has to share with RI. The utility value *u*_*RI*_ shown in row no. 6 is probably too low because (as in the case of the Netherlands) consumers do not see CC and MC as being of equal quality.

These findings suggest that giving German consumers the right to designate the FHF refund to either a SHI or a MCO (the reform proposal) is not likely to oust the {SHI, RI} coalition. The efficiency advantage of MCO may easily fall short of the compensation needed to win Germans over to a MC plan. A choice experiment performed in 2005 found a physician list dressed up by the health insurer would have to be compensated by a reduction in the worker’s contribution amounting to EUR 346 per year (compared to a roughly estimated nationwide average of EUR 3300 at the time); just gatekeeping would require EUR 115 per year. In addition, a status quo preference amounting to EUR 500 would have to be overcome [15]. Therefore, a MCO would have to offer a reduction of up to 26% (equivalent to 13% of total contribution in view of employers’ 50% share) to SHI members to attract them to a “harsh” MC plan and 19% in the case of a “mild” gatekeeping one. Evidence from a major Swiss SHI suggests that prior to controlling for risk-selection effects (which are irrelevant for an individual MCO), the “mild” version of MC is associated with a cost reduction of 31% when combined with a low deductible (which comes closest to the German situation) [23]. Compared to previous estimates compiled by [14], this is on the high side. More importantly, it is doubtful that German MCOs would be permitted to offer reductions in contribution in the required magnitude. Therefore, the {SHI, RI} coalition is likely to continue to dominate, with limited benefit to consumers.

## Conclusion

Recent reforms have promoted MC as a panacea for both lack of consumer sovereignty and rising costs in health care. In Germany, attempts to introduce “soft” variants of MC in the early 2000s were followed by more far-reaching reforms providing the option of HMO-like MC. However, few consumers have been won over to such MC plans as yet.

While existing literature has mainly focused on status quo bias and transaction costs to explain low market shares in early MC environments, this study calls attention to a hitherto little-discussed peculiarity, namely the gatekeeping position of German SHI with respect to MC. In contrast, the Netherlands and Switzerland managed to give consumers full sovereignty as to the choice of MC or CC.

In a first step, this study modeled the gatekeeping behavior of SHI in a bargaining game with MCO. This model successfully reproduces some stylized facts of Germany’s MC experience such as SHI’s emphasis on its head count (see the “State no. 1: no consumer choice” section), the fact that the introduction of MCOs does not coincide with an increasing share of MC (see the “State no. 2: the status quo” section), and the failure of start-up financing to sustainably increase the MC market share (see the “State no. 2: the status quo” section).

Second, the model was extended to capture a reform proposal giving German consumers full sovereignty choosing MC or CC as in the Dutch and Swiss cases. As a main result, the extended model unveils an often overlooked outcome with respect to consumer choice in health care, namely the alliance {SHI, MCO} that yields a zero payoff for the consumer.

In fact, the game is shown to be relatively open a priori with either {SHI, MCO}, {MCO, RI} or {SHI, RI} forming a winning coalition. In order to provide more clarity a simulation study of the game under the reform proposal was performed, which found that whichever coalition forms player SHI loses out and the share of MC increases. In particular, the reform involves a trade-off between opportunity and risk for player MCO who took a backseat role compared to SHI prior to the reform, albeit with a low but secure payoff. Clearly, RI cannot lose starting from zero utility in the pre-reform era. Most importantly, the results show that the presence of independent MCOs is not a prerequisite for a higher share of MC or increased consumer utility as is often argued in political debate.

To find clues for a winning coalition the Dutch and Swiss experience was discussed where, in particular, consumer choice experiments revealed that their acceptance of MC requires a high monetary compensation. While for moderate compensations MCOs may still be in the game, the magnitude of necessary premium reductions found in these experiments seriously compromises the efficiency advantage of MC. In that case, the model turns MCOs into losers predicting the coalition {SHI, RI} with a zero payoff for MCO.

These results have strong implications for German health policy. In particular, the focus on MCOs, intended by the legislator as responsible bodies for MC provision, should be viewed in relation to a more promising variant with SHIs integrating MC production according to Dutch and Swiss experience. In addition, simulation results revealed the possible survival of inefficient MCOs under the reform proposal (see the “Results” section). Put into practice, public funding in terms of a start-up subsidy for German MCOs may have backed the wrong horse.

Of course, these conclusions are derived from an analysis that is subject to a number of limitations. First, the crucial role of provider associations in healthcare reform is neglected, the assumption being that they can always be bought off by the SHI. However, German and Swiss experience suggests this not to be possible without support by the government. Second, this brings in the government as a fourth player who pursues its own objectives such as ensuring re-election, to which the other three players contribute in varying degrees. Third, the construct of a representative insured, while greatly facilitating the analysis, is hardly compatible with the quest for consumer choice regarding health care, which enhances efficiency only if preferences are heterogeneous. However, one insight is likely to be robust: Reforms designed to foster MC may meet with resistance not only from healthcare providers and consumers (a known fact), but also incumbent social health insurers (a hitherto neglected fact).

## Appendix A: list of main symbols

**Table:** 

$\bar N$	Population of insured people
*u*_*SHI*_	Objective function of SHI
*N*_*CC*_	Number of insured under CC
*B*	SHI’s budget for public relations
*π*	Payment received by insurer from the FHF per representative insured
*N*_*MC*_	Number of insured shifted to insurer-owned MC plan
*μ*_*SHI*_	Rent per insured in insurer-owned MC plan
*α*	Weight of conventionally insured in objective function
*β*	Weight of surplus in objective function
$N_{MC}^{*}$	Optimal level of MC (SHI’s perspective)
*μ*_*MCO*_	Rent per insured in MCO-owned MC plan
*p*	Transfer price for insured from SHI to MCO
*N*_*MC*,*D*_	Number of insured that MCO plans to contract with
*N*_*MC*,*S*_	Number of insured that SHI is prepared to transfer to MCO at a price *p*
*N*_*SHI*,*MC*_	Number of insured in SHI’s own MC plan
*u*_*MCO*_	Objective function of MCO
$\bar {c}$	Additional bargaining costs
*ρ*	Parameter of RI’s relative risk aversion
*κ*	Scaling factor
*d*	Threat point
$\mathcal {V}$	Bargaining space
*g*_*i*_	Player *i*’s maximum payoff for a fixed payoff of player *j*
$\bar {\mathcal {V}}_{P} $	Pareto efficient boundary of the bargaining space
*v*	Nash bargaining solution (coordinates)
$\tilde {N}_{{MC}}$	Nash bargaining solution (number of insured in MC)
*d*_*a*_	Alternative threat point
*σ*	Money
*u*_*RI*_	Objective (utility) function of RI
$\bar {N}_{k}$	Number of insured under player *k*’s control
*ω*	Side payment
*N*_*SHI*,*CC*_	Number of insured in CC
*m*	Outside option

## Appendix B: reaction functions of the “State no. 2: the status quo” section

In order to derive (6) and (7) consider the constrained maximization of (4) for some fixed *N*_*MC*,*D*_ subject to

19 $$\begin{array}{*{20}l} 0 & \le N_{SHI,MC} \le \bar N  \end{array} $$

20 $$\begin{array}{*{20}l} 0 & \le N_{MC,S}\le \bar N \end{array} $$

21 $$\begin{array}{*{20}l} N_{SHI,MC}+N_{MC,S} & \le \bar N \end{array} $$

22 $$\begin{array}{*{20}l} N_{MC,S} & \le N_{MC,D}. \end{array} $$

Here, (19) to (21) are straightforward constraints for *N*_*SHI*,*MC*_ and *N*_*MC*,*S*_, whereas (22) follows from the fact that *N*_*MC*,*S*_>*N*_*MC*,*D*_ cannot yield an optimum in (4). To begin with, let *N*_*MC*,*S*_<*N*_*MC*,*D*_, and consider the first order conditions (FOCs) derived from (4), i.e.

23 $$\begin{array}{*{20}l} N_{SHI,MC} & = \bar N \frac{\beta}{\alpha + \beta} - N_{MC,S} \frac{\alpha p + \beta\mu_{SHI}}{(\alpha+ \beta)\mu_{SHI}}  \end{array} $$

24 $$\begin{array}{*{20}l} N_{MC,S} & = \bar N \frac{\beta}{\alpha + \beta} - N_{SHI,MC} \frac{\alpha\mu_{SHI} + \beta p}{(\alpha + \beta)p}.  \end{array} $$

A closer (somewhat lengthy) inspection of (23) and (24) shows that they violate (19) for *N*_*MC*,*S*_<*N*_*MC*,*D*_, and hence, that no interior solution exists. Consequently, one may assume that *N*_*SHI*,*MC*_=0 for *N*_*MC*,*S*_<*N*_*MC*,*D*_ (as choosing the second boundary case, i.e. $N_{SHI,MC}=\bar N$, in (4) clearly cannot yield an optimum). Now, the only candidates for an optimum are

25 $$\begin{array}{*{20}l} N_{SHI,MC} \ge 0 &\text{~and~} N_{MC,S} = N_{MC,D}  \end{array} $$

26 $$\begin{array}{*{20}l} N_{SHI,MC} = 0 &\text{~and~} N_{MC,S} < N_{MC,D}.  \end{array} $$

To derive (6), i.e. the interior solution for *N*_*SHI*,*MC*_, apply (25) to (23) by replacing *N*_*MC*,*S*_ with *N*_*MC*,*D*_ for *N*_*SHI*,*MC*_≥0. To derive the first part of (7) note that *N*_*SHI*,*MC*_≥0 holds for $0\le N_{MC,D} \le N^{*}_{MC}$ in (24) where *N*_*MC*,*S*_ is replaced for *N*_*MC*,*D*_ as in (25). The second part of (7) follows from (26), i.e. replacing *N*_*SHI*,*MC*_=0 in (24).

## Appendix C: the bargaining space of the “State no. 3: flexible prices” section

Consider the constrained maximization of (10) over *N*_*MC*_, *N*_*SHI*,*MC*_ and *p* subject to

27 $$\begin{array}{*{20}l} 0 & \le N_{MC} \le \bar N  \end{array} $$

28 $$\begin{array}{*{20}l} 0 & \le N_{SHI,MC} \le \bar N  \\ N_{MC} + N_{SHI,MC} & \le \bar N  \\ \mu_{SHI} & \le p \le \mu_{MCO}  \end{array} $$

for some fixed $\bar u = u_{MCO,c} \in ~[0, (\mu _{MCO}-\mu _{SHI})\bar N - \kappa \bar c]$, cf. (11). Here, the lower bound for $\bar u$ represents the outside option for MCO, while (11) determines the upper bound of the maximum attainable MCO utility. Rearranging (11) yields

29 $$ p=\mu_{MCO} - \frac{\bar u + \kappa \bar c}{N_{MC}}  $$

such that (28) reads

30 $$ 0\le \frac{\bar u + \kappa \bar c}{\mu_{MCO}-\mu_{SHI}} \le N_{MC}.  $$

Using (29) to replace *p* in (10) yields the two-dimensional problem

31 $$ \begin{aligned} & \max_{\left(N_{MC},N_{SHI,MC}\right)} (\bar N-N_{SHI,MC}-N_{MC})^{\alpha} \\ & \qquad \left(\mu_{MCO}N_{MC} +\mu_{SHI} N_{SHI,MC} - \bar u - (1+\kappa)\bar c\right)^{\beta}. \end{aligned}  $$

The corresponding FOCs read

$$\begin{array}{*{20}l} N_{MC} & = \frac{-N_{SHI,MC}(\mu_{MCO}\beta + \mu_{SHI}\alpha)}{(\alpha + \beta)\mu_{MCO}} \\ &\qquad +\frac{ \alpha (\bar u + \bar c(1+\kappa)) + \beta \bar N \mu_{MCO}}{(\alpha + \beta)\mu_{MCO}} \end{array} $$

where

$$\begin{array}{*{20}l} N_{SHI,MC} & = \frac{\bar N \mu_{MCO} \beta (\mu_{SHI} - \mu_{MCO}) }{-\beta \left(\mu_{MCO}^{2}+\mu_{SHI}^{2}\right)-2\alpha \mu_{MCO}\mu_{SHI}} \\ & \qquad + \frac{(\bar u + \bar c(1+\kappa)) \beta (\mu_{MCO}-\mu_{SHI})}{-\beta \left(\mu_{MCO}^{2}+\mu_{SHI}^{2}\right)-2\alpha \mu_{MCO}\mu_{SHI}}. \end{array} $$

Note that *N*_*MC*_=0 is excluded by the lower bound on (11). Further, for *N*_*MC*_>0 and *N*_*SHI*,*MC*_>0 it follows from the FOCs that $N_{MC} + N_{SHI,MC} =\bar N$ such that (31) takes on the value of zero. One may thus conclude that *N*_*SHI*,*MC*_=0 and *N*_*MC*_>0 define the constrained optimum in (31). It remains to consider (30). From the FOCs one finds that

$$ \dot N_{MC} = \frac{\alpha (\bar u + \bar c(1+\kappa)) + \beta N \mu_{MCO}}{(\alpha + \beta)\mu_{MCO}} $$

if (30) is not binding. This is equivalent to $\bar u \le u^{*}$.

Note that $\dot N_{MC} \le \bar N$ by the upper bound on (11) such that (27) holds. Second, if $\bar u > u^{*}$ then the constraint (30) is binding, and

32 $$ \ddot N_{MC} = \frac{\bar u + \kappa \bar c}{\mu_{MCO}-\mu_{SHI}}  $$

solves the optimization problem (giving rise to some shadow price *λ*≥0 with respect to the constraint (30)). Again, the upper bound on (11) shows that $\ddot N_{MC}\le \bar N$. Finally, *p*=*μ*_*SHI*_ follows from (29) and (32).

## Appendix D: pareto frontiers of the “State no. 4: introducing consumer choice” section

1. *Coalition of SHI and MCO.* If SHI and MCO reach an agreement $\bar N_{RI} =0$ follows from Assumption 2a. Otherwise, $\bar N_{RI} = \bar N$ and *d*=(0,0) by (17) and (18). Both players bargain over a partition $\bar N_{MCO} + \bar N_{SHI} = \bar N$ of the insured.
  Consider two different bargaining situations, depending on the legal environment. In the first, side payments in money between SHI and MCO are not allowed (i.e. *ω*=0 in (17) and (18)), reflecting legislators’ aversion against flows of money between private parties in health care. This makes the shifting of insured the only medium of exchange. Then, following similar steps as in Appendix C the payoff frontier $\bar {\mathcal V}_{P}$ is given by (17) for *ω*=0 where
  $$\begin{array}{*{20}l}{} {}N_{SHI, MC} &= \frac{\alpha}{\alpha+\beta} \frac{\bar c}{\mu_{SHI}} + \frac{\beta}{\alpha + \beta} \left(\bar N - \frac{u_{MCO}+\kappa \bar c}{\mu_{MCO}} \right) \\ {}\bar N_{MCO} &= (u_{MCO} + \kappa \bar c)/\mu_{MCO} \end{array} $$
  and $0 \le u_{MCO} \le \mu _{MCO}\bar N - \kappa \bar c$. In the other legal environment bargaining involves the number of insured as well as a money transfer *ω*≥0. The corresponding efficiency gain corresponds to an outward shift of the Pareto frontier $\bar {\mathcal V}_{P}$ which is now given by (17) for
  $$\begin{array}{*{20}l} \bar N_{MCO} & = \frac{\alpha}{\alpha + \beta} \frac{\bar c + \bar c \kappa + u_{MCO}}{\mu_{MCO}} + \frac{\beta \bar N}{\alpha + \beta}  \\ \omega & = \mu_{MCO}\bar N_{MCO}-\kappa \bar c - u_{MCO}\ge 0 \end{array} $$
  and $0 \le u_{MCO} \le \mu _{MCO}\bar N - \kappa \bar c$. In addition, *N*_*SHI*,*MC*_=0; this is intuitive due to the higher efficiency of MCO’s MC production.
2. *Coalition of MCO and RI.* This coalition against SHI yields $\bar N_{SHI}=0$. Further, in case of disagreement $\bar N_{RI}=\bar N$ holds, implying *d*=(0,0). If MCO and RI come to an agreement, $\bar N_{MCO}=\bar N$ is efficient by (16) and (18). In view of the threat *d* an amount *σ*≥0 will be paid by MCO to compensate RI for cooperation. Put differently, *σ* is the medium of exchange, with the size of the cake by (18) equal to $\mu _{MCO}\bar N - \kappa c$. The Pareto frontier $\bar {\mathcal V}_{P}$ is given by (16) for $\sigma = \bar N \mu _{MCO} - \kappa \bar c - u_{MCO}$ and $0 \le u_{MCO}\le \bar N\mu _{MCO} - \kappa c$.
3. *Coalition of SHI and RI.* Here, $\bar N_{MCO}=0$, and $\bar N_{RI}=\bar N$ when no agreement is reached. Again, this yields *d*=(0,0). Otherwise, efficient bargaining requires $\bar N_{SHI}=\bar N$ where similar to coalition {MCO,RI}, the good traded is a side payment $\sigma \le \bar N\mu _{SHI} - \bar c$ that is requested by RI for cooperating. The Pareto frontier $\bar {\mathcal V}_{P}$ follows from maximization of *u*_*SHI*,4_ over *N*_*SHI*,*MC*_ for a given value $0\le u_{RI}\le (\bar N\mu _{SHI} - \bar c)^{1/\rho }$ which yields $N_{SHI,MC} = (\beta \mu _{SHI}\bar N + \alpha (c + u_{RI}^{\rho }))/((\alpha + \beta)\mu _{SHI})$.

## Endnotes

^1^ Note that the PHI itself may offer a service similar to MC or SC under the reform proposal. We excluded PHI as an additional player in our analysis in the “State no. 4: introducing consumer choice” section, however, as the core results of the model remain unchanged when incorporating PHI as a variant of SHI or MCO. See also [12] for a discussion.

^2^ This assumption is plainly counterfactual; participants in choice experiments from Germany, the Netherlands, and Switzerland were found to require substantial compensation in particular for giving up free physician choice (see [15 *,* 27]). Rather than introducing a separate variable, “Compensation required to render CC and MC equivalent”, the surplus values *μ*_*SHI*_ and *μ*_*MCO*_ are to be understood as being net of such compensation.

^3^ As in the conventional definition of a non-cooperative game, any preplay communication and binding agreements between players are precluded.

^4^ A penalty on the MCO whenever *N*_*MC*,*D*_>*N*_*MC*,*S*_ (amounting to the cost of error in business planning) may be considered in (5) in order to prevent $N_{MC,D} = \bar N$ irrespective of *N*_*MC*,*S*_ in (8).

^5^ Where confusion may arise the notation $\mathcal V_{\{ij\}}$ instead of $\mathcal V$ is used to denote the bargaining space of players *i* and *j*. Note that $\mathcal V$ in Fig. 2 b is not comprehensive, i.e. $x\in \mathcal V$ does not imply $y\in \mathcal V$ for *y*≤*x*. The reason is that utility relates to a number of insured, who cannot be burned as is often assumed e.g. for trades involving money.

^6^ Note that one may set *N*_*SHI*,*MC*_=0 by (13) for all points on $\bar {\mathcal V}_{P}$.

^7^ Recall that Assumption 2a does not necessarily imply provision of CC when choosing SHI since a MC plan is owned by SHI.

^8^ Note that $\bar N_{RI} >0$ represents RI’s threat to break off negotiations. When an agreement is reached, however, $\bar N_{RI}=0$ in connection with a monetary payment *σ*>0 from either MCO or SHI to RI (see Appendix D).

^9^ Applying the standard definition, *x*=(*x*_*i*_,*x*_*j*_,*x*_*k*_) is in the core for the coalition structure {{*i* *j*},{*k*}} if $(x_{i},x_{j})\in \mathcal V_{\{ij\}}$ and no pair can feasibly improve upon their outcome.

^10^ To see this, consider an insurer with *α*≪*β* mainly interested in its budget (not included in Table 1). Then, *N*_*MC*_ will be identical or very similar in states no. 3 and no. 4 whereas *u*_*RI*_ increases. Hence, the latter is fully attributable to consumer sovereignty.
